# Impact of quality certification of multidisciplinary head and neck tumor centers

**DOI:** 10.1186/s12962-021-00273-9

**Published:** 2021-04-07

**Authors:** Ali Modabber, Daniel Schick, Evgeny Goloborodko, Florian Peters, Marius Heitzer, Anna Bock, Kristian Kniha, Frank Hölzle, Elke M. Schreiber, Stephan Christian Möhlhenrich

**Affiliations:** 1 Department of Oral, Maxillofacial and Facial Plastic Surgery, Medical Faculty, University Hospital RWTH Aachen, Pauwelsstr. 30, 52074 Aachen, Germany; 2 Department of Intensive Care Medicine, Medical Faculty, University Hospital RWTH Aachen, Aachen, Germany; 3 University Hospital of Johannes Gutenberg University Mainz, Mainz, Germany; 4 Department of Orthodontics, University of Witten/Herdecke, Witten, Germany

**Keywords:** DRG, Certification, Tumor board, Cost, Efficiency, Head‐Neck, Maxillofacial, Oropharyngeal, Multidisciplinary tumor center

## Abstract

**Background:**

Certification of multidisciplinary tumor centers is nowadays seen as the gold standard in modern oncological therapy for optimization and realization of guideline-based therapy and better outcomes. Single cases are reimbursed based on diagnosis-related groups (DRG). We aimed to review efficiency, cost analysis, and profitability following a certification.

**Methods:**

Tumor board certification at the university hospital Aachen was implemented in 2013. We compared 1251 cases of oropharyngeal cancer treated from 2008 to 2017 before and after certification. For this purpose, several patient characteristics, surgery, and stay-related constants, as well as expenses and reimbursement heights were analyzed statistically.

**Results:**

Following certification, the total case and patient number, surgery duration, hours of mechanical ventilation, case mix index points, DRG reimbursements as well as the costs increased significantly, whereas days of intensive care unit, amount of blood transfusions, patient clinical complexity level (PCCL) and the overall stay were significantly lowered. No changes were observed for the patient’s age and gender distribution. Also, the predetermined stay duration stayed constant.

**Conclusions:**

Certification of head-neck tumor centers causes a concentration of more complex cases requiring higher surgical efforts, which can be processed more efficiently due to a higher level of professionalism. Despite their benefits in cancer care, without compensation, centers may be struggling to cover their expenses in a system, which continuously underestimates them.

## Introduction

To face challenges in times of rising medical expenses and to guarantee transparency and efficiency, the government of Germany introduced diagnosis-related groups (DRG) system in 2000, which aimed to sort hospital cases into medical and economical comparable groups [1, 2]. The final DRG takes the admission diagnosis, treatment procedures, and comorbidities, which are represented by the patient clinical complexity level (PCCL), into account and is further allocated into specific cost weight groups to generate the reimbursement height. The sum of cost weights for a specific time frame results in the case-mix, which divided by the case number produces the case mix index (CMI), an indicator of the average case severity [3–5]. The introduction of DRG has ever been a controversial topic. Advantages like increased efficiency, transparency, and reduced average length of stay conflict with financial benefits from earlier discharges [6–8].

Today interdisciplinary treatment concepts are an essential part of modern oncological therapy for optimization and realization of guideline-based therapy and better outcomes [9]. Certification processes aim to ensure transparent quality guidelines that serve the affected patient as a landmark while searching for the best possible oncological support [10]. Oropharyngeal carcinoma account for 3% of all cancer worldwide and are mostly diagnosed in older patients in combination with tobacco and alcohol abuse, but also in association with HPV and younger adults. Head neck tumor center certification has been implemented in 2010 and has been rising in numbers ever since. In 2013 the department of oral and maxillofacial surgery at the University Hospital Aachen has been certified by the German Cancer Society carried out by OnkoZert. This implies a network of qualified, interdisciplinary, trans-sectoral facilities and if so, extending over locations, which displays the entire medical care for tumor patients [10, 11].

This study aimed to reveal changes in the financial outcome, patient complexity, stay duration, and patient characteristics since the implementation of certification, to conclude the practicability and affordability of the current situation.

## Materials and methods

### Data collection

This retrospective study includes a total of 1251 cases of oropharyngeal cancer. Inclusion criteria for patients were met under the following conditions: oropharyngeal carcinoma, discharge between 2008 and 2017, presented in an multidisciplinary team (MDT) conference and tumor board, and the patient treated at the department of oral and maxillofacial surgery at the University Hospital Aachen from 2008 to 2017. Also, reconstructive and rehabilitative interventions were included. Patients from other clinics, without proof of primary tumor manifestations, other tumor locations, outpatient treatments, or refusal of intervention were excluded. The data of this retrospective study were exported from the hospital’s internal information system Medico (Siemens, Munich, Germany) into Microsoft Excel 2011 (Microsoft Corporation, Redmond, WA, USA). The average costs for each DRG derive from the data of the “organization of German university hospitals” (Verband der Universitätsklinika Deutschlands e.V.) on the base of reference hospitals without a statement to the actual database of the university hospital Aachen.

### Operating figure

The following operating figures of participating patients were used for further calculations and interpretations: Total case number, age, sex, length of stay, average length of stay, case mix index points, costs, DRG reimbursement, PCCL, surgery duration, days of intensive care unit, hours of ventilation and amount of blood transfusions. The PCCL value is calculated from the patient’s comorbidities, representing their cumulative severity. Thereby increased financial burden can be respected and reimbursement was adopted for its highest value. In 2016 the maximum PCCL was increased to 6 points. To guarantee reproducibility values of 5 and 6 were depicted as 4. The calculation formula was also modified in 2014/15 [12].

### Statistics

Quantitative data are shown as mean *± SEM*. The diagrams and statistical calculations were performed with PRISM 7.0 (GraphPad Software, La Jolla, USA). Data were tested for Gaussian distribution using the Shapiro-Wilk test and homoscedasticity using the F-test. Afterward, a student’s t-test or one-way ANOVA with FDR correction was performed for the final statistical evaluation. Analysis of data sets, which did not meet the criteria was consequently calculated using the equivalent non-parametric test. Fisher’s exact test was used for categorical variables. A p-value < 0.05 was always considered significant. A detailed description of the statistics can be found underneath the corresponding figure.

## Results

**Fig. 1:**
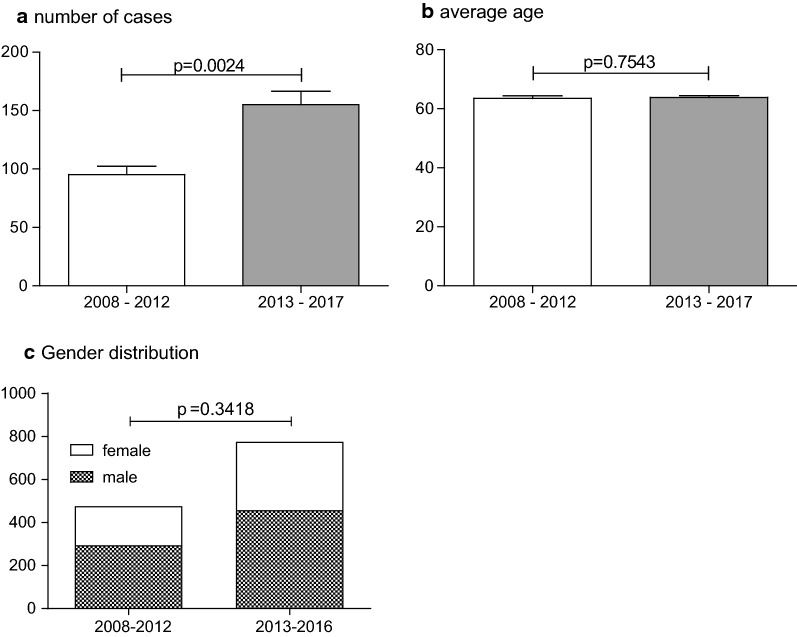
Comparison of general characteristics from 2008–2012 to 2013–2017. **a** Number of cases per year was tested parametrically with student’s t-test. Data are shown as mean ± SEM. **b** The average age was tested nonparametrically with Mann-Whitney U test. Data are shown as mean ± SEM. **c** Gender distribution changes was assessed with Fisher’s exact test. Data are shown as absolute count

To assess the impact of this quality certification, cases before (2008–2012) and after the certification (2013–2017) were compared for multiple factors. In Fig. 1 general characteristics are compared. Following the certification, a significant increase in the number of treated cases can be seen from an average of 95.2 *± 7.24* before to 155 *± 11.63* cases afterward per year (p = 0.0024, Fig. 1a). Regarding the average age no significant difference can be found before and after (63.53 *± 0.81* vs. 63.83 *± 0.57* years; p = 0.7543, Fig. 1b). Also, the ratio between males and females did not change significantly, despite the general increase in treated patients. Before the certification 294 male and 182 female patients underwent a treatment, which changed to 457 male and 318 female patients afterward (p = 0.3418, Fig. 1c).

**Fig. 2:**
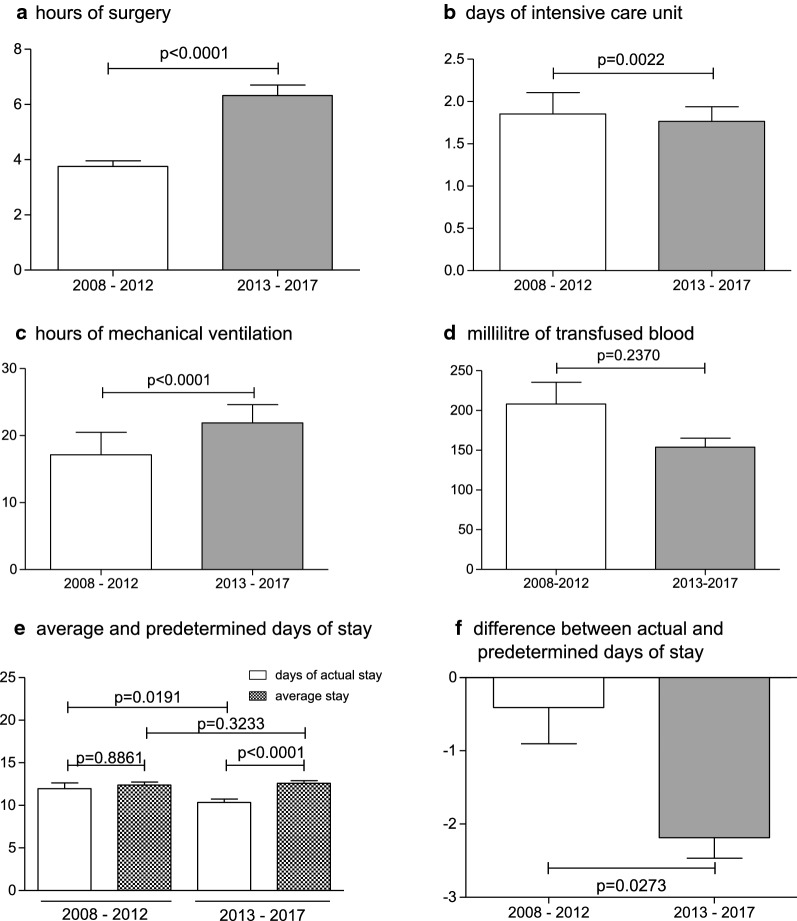
Comparison of intervention and stay duration from 2008–2012 to 2013–2017. **a**–**d**, **f** Hours of surgery, days at the intensive care unit, hours of mechanical ventilation, total amount of transfused blood products (milliliter) and the difference between actual and predetermined days of stay were test nonparametric with Mann-Whitney U test. **e** Comparison of average and actual stay were test parametric with one-way ANOVA with post-hoc Tukey for multiple comparisons. Data are shown as mean ± SEM

Figure 2 regards the characteristics of the general intervention and overall stay duration also in comparison to the default average length of stay, which is predetermined for each DRG. Surgeons operated in average significantly longer for each case after the implementation of the certification with 3 h 45 min *± 12 *min per case before and 6 h 19 min *± 23 *min afterward (p < 0.0001, Fig. 2a). The average case length at the intensive care unit postoperatively could be decreased from 1.85 *± 0.25* days to 1.77 *± 0.17* days (p = 0.0022, Fig. 2b), whereas the hours of mechanical ventilation were increased from 17 h 08 min *± 3.34 h* to 21 h 52 min ± *2.73 h* (p < 0.0001, Fig. 2c). The need for blood transfusion products decreased from 206 *± 27* ml to 154 ± *11 *ml but did not reach significance (Fig. 2d). Regarding the length of the overall stay, patients could be discharged earlier after 10.34 *± 0.40* days following the certification instead of 11.98 *± 0.65* days before (p = 0.0191, Fig. 2e), whereas the expected and predetermined duration by DRG stayed constant (12.39 *± 0.36* days before, 12.60 *± 0.31* days after; p = 0.3233, Fig. 2e). Following these observations, patients could be significantly 2.26 ± *0.28* days earlier discharged than predetermined by DRG after the implementation of certification versus 0.41 ± *0.50* days before (p = 0.0273, Fig. 2f).

**Fig. 3:**
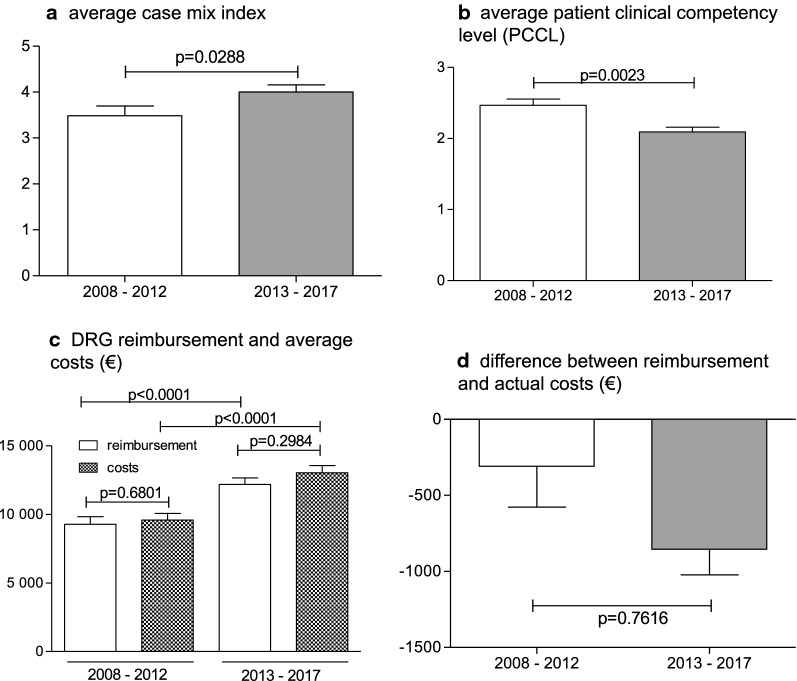
Comparison of patient complexity and financial records from 2008–2012 to 2013–2017. **a**, **b**, **d**: Average case mix index, average patient clinical competency level (PCCL) and the difference between DRG reimbursement and actual costs were tested nonparametric with Mann-Whitney U test. **c** Comparison of DRG reimbursement and average cost were assessed parametric with one-way ANOVA and post-hoc Tukey for multiple comparisons. Data are shown as mean ± SEM

Figure 3 concerns the overall complexity and financial questions. The average case mix index increased significantly after certification from 3.49 *± 0.21* to 4.00 *± 0.15* (p = 0.0288, Fig. 3a), whereas the patient clinical complexity level per patient significantly decreased from 2.47 *± 0.09* to 2.09 *± 0.07* (p = 0.0023, Fig. 3b). Every DRG has a predetermined amount, which is reimbursed for the specific case. This value has significantly increased after certification from 9,270€ *± 556.40€* to 12,183€ *± 467.80€* per case (p < 0.0001, Fig. 3c). Accordingly, the costs for the hospital increased from 9,578€ *± 492.20€* to 13,037€ *± 523.60€* (p < 0.0001, Fig. 3c). The deficit for the hospital, therefore, was 308€ *± 268.70€* before and 854€ *± 169.10€* afterward, but without reaching a significant difference (Fig. 3d).

## Discussion

The therapy of oropharyngeal carcinoma includes resection of the primary tumor with neck dissection and reconstruction. Chemotherapy or radiotherapy might be necessary for advanced stages [13]. MDT approaches for head-neck tumors have been shown in various retrospective and prospective studies to refine disease staging, treatment plans and to increase survival rates [14–19]. Also, they could enable faster treatment and shorter time of hospitalization [20–22]. Wheeles et al. could prove in a setting of patients with head-neck tumors, that approximately 27 % of the patients revealed changes in tumor diagnosis, stage, or treatment plans after undergoing an MDT discussion. Other studies confirmed these results [23, 24]. Loevner et al. discovered changes in image interpretation in 41 % of patients after MDT reevaluation [25].

Following its certification in the year 2013 as an interdisciplinary centrum for head-neck tumors, the university hospital Aachen revealed a significant increase in cases. Those results are not surprising considering that patients are aiming especially for certified centers hoping to receive the highest standard of quality in terms of medical performance but also other factors like psychological assistance or case management. Additionally, every certification may lead to an increase in popularity and advertisement.

In this study we tried to evaluate, whether the overall case complexity has changed since certification, assuming that more severe cases are treated in certified centers to guarantee the absolute best outcome. The PCCL value, which summarizes the secondary diseases, and CMI, as overall average case severity, is most likely reflecting the complexity [26]. Although these values may indicate the case complexity, a full medical comparison would still have to include TNM classification, former surgeries, and radiotherapy, which do not directly influence the CMI but increase surgery duration. Nevertheless, the CMI encountered a significant increase, which is accompanied by the hours of surgery and hours of ventilation, which reflects a higher surgical effort and case complexity.

On contrary, the PCCL decreased significantly, which however must be regarded cautiously because it has faced several modifications throughout the study. Values above 4 in 2016 and 2017 were consequently downsized to 4. All this led to a general flattening of the PCCL incline curve. Additionally, patients rested significantly less time under intensive care surveillance. Accordingly, the overall days of stay in the hospital decreased significantly. This shortness in stay duration can be explained by an increasing cost pressure for hospitals nowadays, a higher level of specialization, professionalism, and discharge management, a higher frequency of similar case encounters, but also a higher case number, which allows a more sophisticated postoperative handling [27, 28]. The better case planning in terms of a preoperative diagnostic and staging phase, but also a prompt application for rehabilitation are all quality features, which are implemented in the certification. Kelly et al. could prove accordingly, that patients undergoing MDT could be discharged more than a week earlier, suspecting a decrease in the overall waiting time as the cause [20].

Another important aspect of this study was to evaluate possible financial changes following certification. The average deficit between costs and reimbursement per case increased after 2012 without reaching significance (308€ vs. 854€, p = 0.7616). The DRG reimbursement is calculated by multiplying CMI and the state-wide base rate (“Landesbasisfallwert”), which is negotiated retrospectively every year and increased continuously over the years. Since the relation between reimbursement and the state-wide base rate is reflected by the CMI, we reason, that the increased reimbursement costs can be explained by an increasing CMI [29, 30]. Due to the retrospective character of this study, an actual cost determination of each patient case at the university hospital Aachen was not possible and we had to refer to average DRG costs derived from the German association of university hospitals. Since the database of reference clinics was not published by the association, cost analysis should be regarded cautiously. Nevertheless, the method of surgical intervention did not change relevantly over time and therefore did not impact cost development. So far our study group comprehends additional charges only for few occasions: Patient-specific implants (plates), which were not part of this collective, and prolonged intensive care unit stays. Our analysis did not include those center surcharges and further compensations.

Derived from our data and other studies, we could observe that it was difficult for hospitals to cover all expenses only by DRG reimbursement in this group of patients over the years [31]. Although the general stay duration decreased, costs climbed significantly, because the last days of stay are in general linked to the lowest costs. Also, regarding the DRG-reimbursement curve, compensation costs are equal between the minimal and maximal marginal stay duration, which therefore hardly enables a cost reduction. On the other hand, the general cost increase can be well explained by the higher degree of patient complexity and longer operation time. A huge disadvantage of the DRG compensation is its retrospective approach, which adapts the reimbursement height annually to the costs of the previous years [27, 32]. The broader implementation of additional charges (“Zusatzentgelte”) or new examination and treatment methods (NUB) for covering expensive procedures and products could facilitate the reimbursement of high-priced medicine and foster innovations, although there is little evidence so far [33, 34]. Performance-based remuneration could additionally motivate to improve the efforts undertaken. This could include e.g., overall survival rate, degree of relapse, hospital stay length, or changes in life quality indexes postoperatively, but should always be related to the PCCL, CMI, and further aspects as former surgeries or tumor stage. Lerch et al. proposed alternatively to separate the DRG System between a University Hospital U-DRG-System and a general G-DRG-System, thereby creating an independent system for more complex and costlier cases [31]. Additionally, the process of certification should not be underestimated in terms of its accompanying establishment fees. To enable and maintain such quality, recertification, and a lot of personal and financial resources (case management, psych oncological care, interdisciplinary tumor conferences) are required. Kelly et al. described, that any additional administrative costs could be compensated by the savings, which can be achieved by a better patient evaluation [20]. Some studies are doubting the potential benefits of MDT, declaring them as too costly and inefficiently, assuming that MDT should be rather be reserved for complex cases [35–37]. Other studies could, on contrary, prove that during MDT meetings most of the time was spent on complex and advanced cases, while simple cases were finished off quickly [38, 39]. However, cost analysis evaluation stays until today scarcely investigated [40].

## Conclusions

Our data show, that it may be difficult for hospitals to cover expenses only by DRG reimbursement without further compensations. While the process of certification is primarily aiming to improve the quality, the reimbursement system only considers the number of procedures, ignoring the tumor stage and prognosis, which decides the level of compensation in the end. Regarding the obvious advantages of certified tumor centers for the patient’s benefits, in the future the government will be obliged to compensate for the cost, more appreciating the efforts already undertaken. One possibility could be to add success-dependent awards for centers, to further improve therapy results and cost-efficiency.

## Data Availability

The datasets used and/or analyzed during the current study are available from the corresponding author on reasonable request.

## Abbreviations

DRG: Diagnosis-related groups
PCCL: Patient clinical complexity level
CMI: Case-mix index
MDT: Multidisciplinary team
TNM: Classification of Malignant Tumors
